# Distribution of Main Bioactive Compounds from Saffron Species as a Function of Infusion Temperature and Time in an Oil/Water System

**DOI:** 10.3390/molecules29133080

**Published:** 2024-06-28

**Authors:** Inmaculada Criado-Navarro, Carlos Augusto Ledesma-Escobar, Pedro Pérez-Juan, Feliciano Priego-Capote

**Affiliations:** 1 Department of Analytical Chemistry, University of Córdoba, 14071 Córdoba, Spain; inma.c.n@hotmail.es (I.C.-N.); z32leesc@uco.es (C.A.L.-E.); 2 Chemical Institute for Energy and Environment (iQUEMA), University of Córdoba, 14014 Córdoba, Spain; 3 Maimónides Institute of Biomedical Research (IMIBIC), Reina Sofía University Hospital, University of Córdoba, 14004 Córdoba, Spain; 4 CIBER of Frailty and Healthy Ageing (CIBERFES), Carlos III Health Institute, 28029 Madrid, Spain; 5 Azafrán de La Mancha Protected Designation of Origin Regulatory Council, 45720 Camuñas, Spain

**Keywords:** saffron, crocins, safranal, picrocrocin, bioactive compounds, mass spectrometry

## Abstract

Most research on saffron has focused on its composition and beneficial effects, while the culinary perspective to enhance its gastronomic potential remains unexplored. This study aims to define the transfer of the main compounds responsible for color, flavor, and aromatic properties, evaluating three critical variables: temperature (60 °C, 80 °C and 100 °C), infusion time (ranging from 10 to 30 min), and the composition of the medium (water, oil, and water/oil). Samples were analyzed using the LC-QTOF MS/MS and ISO 3632-1:2011 methods. The major compounds were crocins, including trans-crocin and picrocrocin. Among the flavonoids, kaempferol 3-O-sophoroside stands out. Regarding extraction conditions, crocins, glycoside flavonoids, and picrocrocin were enhanced in water, the former in 100% water and at low temperatures, while picrocrocin proved to be the most stable compound with extraction favored at high temperatures. The variable with the greatest incidence of picrocrocin isolation seemed to be the concentration of water since water/oil compositions reported higher concentrations. Safranal and kaempferol were enriched in the oil phase and at lower temperatures. This study provides a chemical interpretation for the appropriate gastronomic use of saffron according to its versatility. Finally, the determination of safranal using the ISO method did not correlate with that obtained using chromatography.

## 1. Introduction

Saffron is a spice obtained through dehydrating the stigmas isolated from the flower of *Crocus sativus* L. flower, and is cultivated in Iran, Spain, India, Italy, Afghanistan, Azerbaijan, UAE, Turkey, France, Egypt, Switzerland, Israel, Greece, China, Japan, Iraq, and recently in Australia (Tasmania) [1]. Specifically, saffron is a product that is closely linked to Mediterranean culture, as evidenced by the four Protected Designations of Origin (PDO) recognized in this area within the European Union. In Spain, the PDO Saffron of La Mancha has fully inherited the quality prestige of Spanish saffron in the market [2].

The applications of saffron in food, medicine, perfumes, and cosmetics have been documented by several authors. It is a very ancient spice that has been used for almost four millennia as an additive in food elaboration due to its coloring, flavoring, and aromatic capacity [3,4,5]. In addition, its special properties have been appreciated as a textile and wool dye, incense, beauty product, and perfume, and in the treatment of medical indications [6]. Most of the medicinal effects of saffron are related to its strong antioxidant activity, which is mainly attributed to crocins, crocetin, and safranal [7]. Beneficial effects such as anti-inflammatory, antileukemic, antihypertensive, neuroprotective, antidiabetic, and antidepressant effects, among others, have been documented by many authors [8,9,10,11,12,13].

Saffron’s chemical composition is complex. This spice has primary metabolites that are ubiquitous in nature, such as carbohydrates, minerals, fats, vitamins, amino acids, and proteins. Many secondary metabolites are also present, such as carotenoids, monoterpenes, and flavonoids, including anthocyanins [1]. The quality of saffron is evaluated chemically by determining three major secondary metabolites: namely, crocin (C_44_H_64_O_24_, water-soluble crocetin esters), picrocrocin (C_16_H_26_O_7_, monoterpene glycoside, the precursor of safranal) and safranal (C_10_H_14_O), which are responsible for color, flavor, and aroma, respectively [14]. The color comes from a mixture of the cis- and trans-isomers of the glycosylated esters of crocetin with glucose, gentiobiose, or neapolitan. The bitter taste of saffron is mainly due to picrocrocin, although flavonoids may also contribute to this taste and astringency sensation. Finally, the characteristic aroma of this spice is related to safranal, although a significant number of other volatile components contribute to the complex aroma [15,16,17].

The quality of saffron is determined according to the 3632-1:2011 ISO standard [18], which distinguishes three categories based on the following physicochemical parameters: presence of floral residues, moisture and volatile components, ash content, coloring power, bittering power, and aromatic power. ISO 3632-1:2011 proposes the quantification using the photometry of picrocrocin, safranal, and crocins at a maximum wavelength of 257, 330, and 440 nm, respectively [18]. However, some authors noted that crocins have overlapping absorption spectra between 250 and 470 nm. Another group of compounds that could interfere with these measurements to determine saffron quality are kaempferol derivatives, which absorb UV light at 264 and 344 nm. Thus, overlapping causes quantification errors and limitations in this method [19,20]. In the search for more efficient methods, liquid chromatography (LC) has been proposed as a separation technique to increase the resolution capability. Several studies have described the identification and detection of saffron metabolites using LC-based methods including safranal, crocins, picrocrocin, and kaempferol derivatives. These methods show high performance when mass spectrometry (MS) is coupled with LC to improve quantification and facilitate compound identification through structural elucidation [21,22,23,24,25].

The literature on the culinary perspective and practical use of saffron to enhance its gastronomic potential is scarce. Rodriguez-Neira et al. showed that an aqueous infusion of saffron for more than 10 min at 100 °C resulted in a clear decrease in color and aroma without significantly affecting the bitter taste [26]. However, there are no studies that simulate different conventional culinary phases, such as water/oil with temperature variations, which could provide chemical information on the distribution of the main chemical families of saffron. This study aims to define the transfer of chemical components responsible for the aroma, color, and flavor of saffron to a liquid phase to improve its applicability. Temperature and infusion time were evaluated as variables with a critical influence on the solubility of these components, while the composition of the liquid phase was studied to quantify the transfer according to the polarity of the medium. The chemical results would allow the interpretation of the optimal conditions to exploit the color, taste, and aroma power of saffron in culinary applications. For this purpose, we combined the analysis of processed samples using an LC-QTOF MS/MS method and the ISO 3632-1:2011 standard.

## 2. Results and Discussion

Saffron is mainly used in the agro-food industry due to its organoleptic properties related to its coloring and flavoring properties. The main compounds responsible for these properties are crocins, safranal, and picrocrocin, responsible for saffron’s color, aroma, and bitter taste, respectively [27]. Although spices have traditionally been used for food preparations to improve flavor and taste, they are also often used as antioxidant food supplements. The antioxidant activity of saffron is mainly explained by the presence of crocin and crocetin, and the synergistic effect of all bioactive constituents [28,29]. Therefore, this study aimed to define the transfer of the mentioned bioactive compounds to the liquid phases (water or oil) by simulating cooking conditions with temperatures between 60 and 100 °C and infusion times of 10, 20, and 30 min.

### 2.1. Crocins Profiling

Crocins are the main compounds responsible for the coloring power of saffron. The total content of crocins in saffron is approximately 16–28% [27]. In our analyses, we identified crocin, crocin-2, and crocin-3 (Figure S1), with the discrimination of cis- and trans-isomers using chromatographic separation. All of them yielded two distinct product ions at *m*/*z* 327.157 and 283.167.

**Figure 1:**
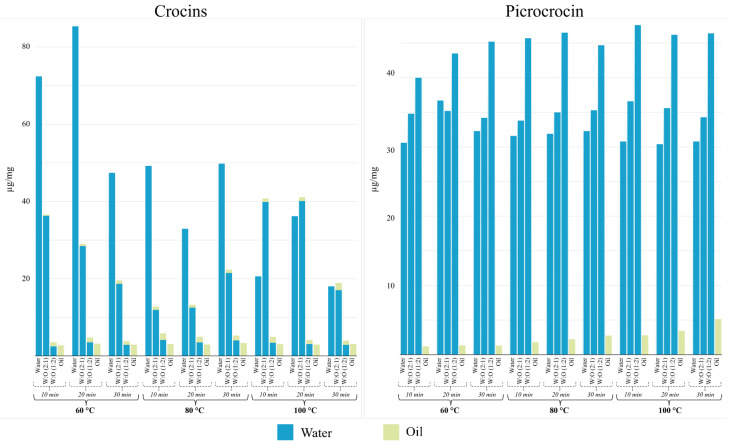
Concentration of crocins and picrocrocin (µg/mg) as a function of infusion time, extraction medium, and temperature. Concentration variability was always below 15%.

Due to the polar nature of crocins, they were mostly detected in the water assays. The total crocin content was calculated by the sum of the concentrations of all crocin species determined via LC-QTOF MS/MS analysis using the calibration curve. In the experiments involving a biphasic system of oil/water, the total crocin content was estimated by calculating the amount of the monitored species in the two phases analyzed independently. The influence of time and temperature variables on the total content of crocins is shown in Figure 1. Crocins were detected at the highest concentration, 85.4 µg/mg, in water at 60 °C and after 20 min of infusion. Crocins have low stability and lose their functionality when exposed to heat, oxygen, light absorption, acidic environments, and/or due to the presence of additives [24]. In agreement with other studies, we verified that the enrichment of crocins in water decreased at higher temperatures, but also by extending the infusion time to 30 min [30]. Enrichment in 2:1 (*v*/*v*) water/oil at 100 °C for 10 and 20 min resulted in concentrations of 20.6 µg/mg and 36.2 µg/mg, respectively. In the case of 1:2 (*v*/*v*) water/oil and pure oil, the extracts resulted in concentrations always below 5.8 µg/mg and 3.1 µg/mg, respectively. Thus, the coloring power of saffron is favored in aqueous mediums at not excessively high temperatures (60 °C) and with an intermediate infusion time (20 min). These results reported that longer times could lead to the hydrolysis of crocins. This description was consistent for individual crocins (Figure S2). Thus, trans-isomers dominated the crocin profile, especially trans-crocin followed by trans-crocin-2. Some studies suggested the higher stability of the trans-isomer; therefore, the isomerization from trans to cis is enhanced by both, heat, and oil addition [31,32].

### 2.2. Picrocrocin

Picrocrocin, the glycoside derivative of safranal (Figure S3), is primarily responsible for the flavor of saffron and is the second most abundant component (by weight) after crocins, accounting for approximately 1 to 13% dry matter [1]. Picrocrocin is a molecular marker of saffron, identified only in the genus *Crocus*, with *Crocus sativus* L. being the only edible species [7].

The case of picrocrocin differs from that of crocins since the former was clearly detected at higher concentrations using 1:2 (*v*/*v*) water/oil as a medium. In addition, temperature favored the accumulation of picrocrocin. Figure 1 shows that picrocrocin is easily extracted and, practically, there are virtually no significant differences (*p*-value > 0.05) associated with temperature. Thus, 100 °C was required to reach the maximum concentration in 10 min, 47.6 µg/mg, while similar concentrations were obtained at 60 and 80 °C. The variable with the greatest incidence of picrocrocin isolation seems to be the concentration of water since water/oil compositions reported higher concentrations of picrocrocin than water. Therefore, picrocrocin is efficiently transferred to the aqueous medium and the extraction efficiency did not depend on the volume of water as the extraction equilibrium is quickly reached. When the extractant is 100% oil, we observed that the concentration in this medium increased with increasing temperature and extraction time. However, the maximum concentration of picrocrocin in oil was 5.13 µg/mg at 100 °C in 30 min.

### 2.3. Safranal

**Figure 2:**
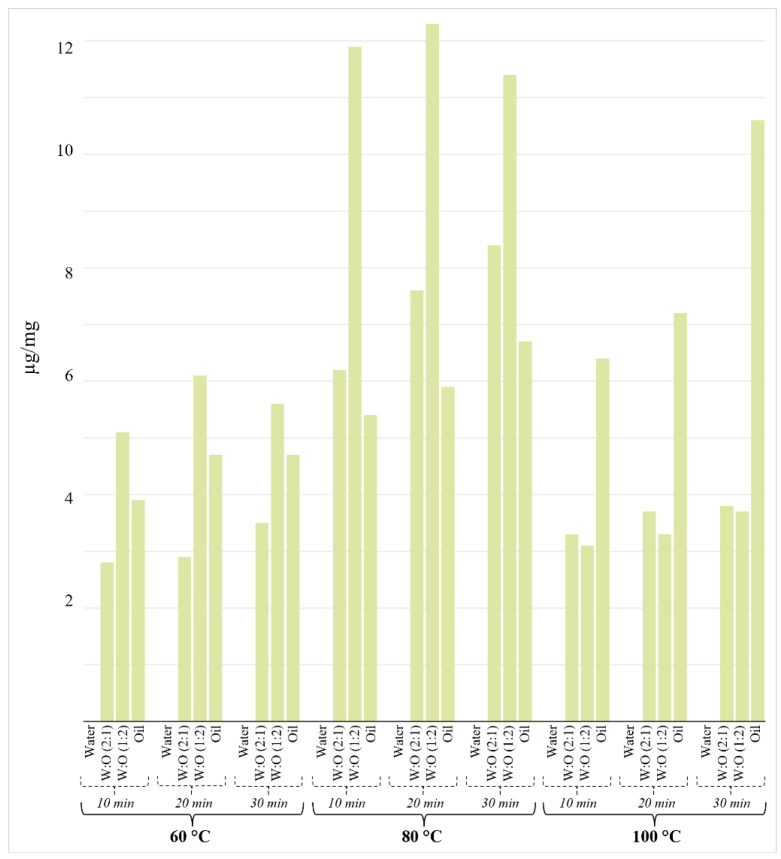
Concentration of safranal (µg/mg) as a function of infusion time, extraction medium, and temperature. Concentration variability was always below 15%.

Safranal is directly related to the aroma of saffron and makes up about 30 to 70% of saffron essential oil and about 0.001 to 0.006% of dry matter, and its concentration in saffron is highly dependent on both drying and storage conditions. Safranal is formed by the hydrolysis of picrocrocin by β-glucosidase action (Figure S3). This chemical change affects the polarity as compared to picrocrocin and crocins. Thus, safranal is detected at trace levels in 100% water. As shown in Figure 2, safranal is easily extracted at 80 °C in 1:2 (*v*/*v*) water/oil regardless of the infusion time, reaching a maximum of 12.3 μg/mg at 20 min. However, the experiments at 100 °C in the presence of water showed a significant decrease in this compound, while a higher concentration was found in the 100% oil phase. In the oil phase, a clear growth trend was observed with increasing temperature and time.

### 2.4. Flavonols

**Figure 3:**
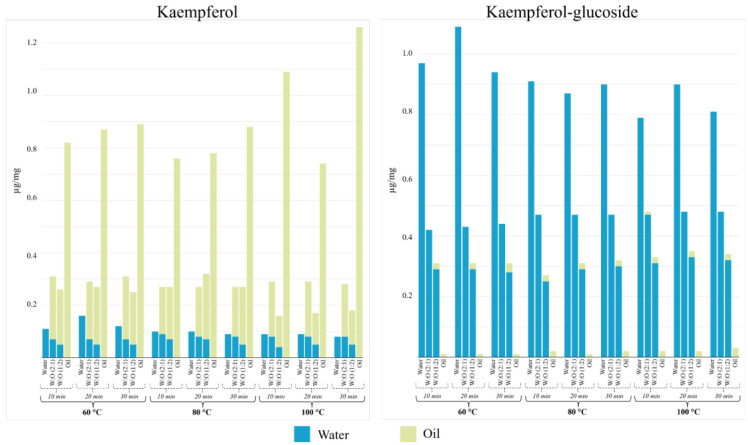
Concentration of kaempferol aglycone and kaempferol glucoside (µg/mg) as a function of infusion time, extraction medium, and temperature. Concentration variability was always below 15%.

Flavonols are a family of compounds belonging to the large group of flavonoids. Aglycone forms of kaempferol and quercetin, as well as some glycosylated derivatives of them, were detected in the extracts obtained from saffron. In particular, when comparing experimental situations, di- and triglucoside derivatives were found in high concentrations with significant differences (*p*-value < 0.05). Kaempferol was mostly extracted in 100% oil, with variable behavior when time and temperature were modified (Figure 3 and Figure S4); the maximum concentration of kaempferol (1.26 μg/mg) was obtained at 100 °C and 30 min of extraction.

Glycoside derivatives showed the opposite response to kaempferol when quantitatively transferred to 100% water (Figure 3 and Figure S4). The most favorable conditions were obtained at 60 °C and 20 min in water in all cases. Quantitative differences were found among them, with the disaccharide conjugate being found at a higher concentration. This result agreed with previously published data [32] and confirmed that kaempferol disaccharide, especially kaempferol 3-O-sophoroside, was the main saffron flavonoid (about 55% of the total flavonol content). In addition, Carmona et al. [33] indicated that kaempferol 3-O-sophoroside content could differentiate the geographical origin of saffron samples.

The case of quercetin and quercetin 3,7-diglucoside coincided with that of kaempferol since they were clearly detected at higher concentrations in pure water at 60 °C. On the other hand, another quercetin derivative, moracetin, was better extracted in the oil phase at temperatures above 80 °C (Figure S5). Quantitatively, the results confirmed that kaempferol glucosides represent 90.3% of the total flavonol content of *Crocus sativus* L. flowers, whereas quercetin and its derivatives represent only 9.7% [33]. It is remarkable that despite the presence of flavonoids in the saffron species, their concentration is too low regarding healthy benefits in humans since (i) their consumption is sporadic and (ii) only a few mg of them is used for cooking each time. Therefore, it could be important in the self-preservation of saffron due to its antioxidant capacity.

### 2.5. Extraction Efficiency at Low Temperature

**Figure 4:**
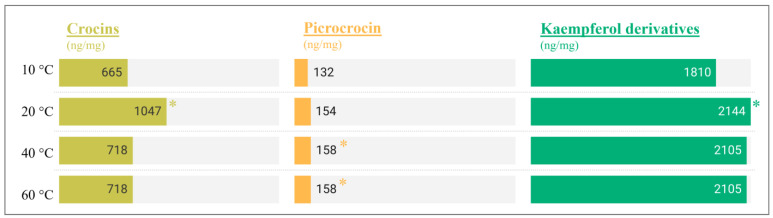
Concentration of crocins, picrocrocin, and kaempferol derivatives (ng/mg) in infusions prepared at temperature from 10 to 60 °C. The asterisk indicates significant differences among infusion temperatures (*p* < 0.05).

The optimal extraction of the monitored compounds was reported in most cases at 60 °C, the lowest temperature tested. Therefore, a complementary study was carried out to investigate whether the temperature is an essential factor by testing the transfer of compounds at 10, 20, and 40 °C. Water was used as a medium and the infusion time was set to 20 min. Figure 4 shows the results obtained for crocins, picrocrocin, and kaempferol disaccharide. The concentration measured for safranal and quercetin at low temperatures was very low, confirming that these compounds are preferentially transferred in an oily phase or at higher temperatures.

The transfer of crocins was favored at 20 and 40 °C compared to 60 °C, which could be explained by stability. A similar reaction was found for kaempferol disaccharide. On the other hand, picrocrocin proved to be the most stable compound with no significant differences (*p*-value > 0.05) when comparing the tests at temperatures from 10 to 60 °C.

Therefore, heating is not necessary to extract crocins to increase the coloring power and bioactive compounds such as kaempferol disaccharide. On the other hand, the temperature is important to obtain a more intense bitterness associated with picrocrocin, since the maximum concentration was obtained at 80 °C.

### 2.6. Comparative Evaluation of the ISO 3632-1:2011 Method and LC–MS/MS

The samples were additionally analyzed using the ISO 3632-1:2011 method (Figure S6). The results for crocins and picrocrocin agreed with those obtained using LC–MS/MS analysis for the extracts. However, the measurement of safranal showed differences compared to LC–MS/MS. The photometric measurement showed that the enrichment was higher in 2:1 (*v*/*v*) water/oil, whereas in the chromatographic analysis, the optimal phase was 1:2 (*v*/*v*) water/oil. There seems to be an overestimation in the determination of safranal because both crocins and crocetin have overlapping absorption bands with safranal and picrocrocin [24]. This situation may explain why the ISO 3632-1:2011 method (2011) does not distinguish saffron categories based on safranal content [34]. In addition, some studies showed a poor correlation between the total crocin content obtained using the ISO method and using LC–MS/MS [31,35].

## 3. Materials and Methods

### 3.1. Samples, Reagents, and Solvents

A pool of 25 g of saffron was provided for this study by the PDO La Mancha Saffron (Camuñas, Toledo, Spain). As part of the sample preparation, saffron was ground for homogenization. The solvents used for sample preparation were LC-grade ethanol from Scharlab (Barcelona, Spain). The MS-grade formic acid used as an ionization agent and acetonitrile, used to prepare the chromatographic mobile phase, were from Fisher Scientific (Madrid, Spain). Deionized water (18 MΩ·cm) was obtained from a Milli-Q water purification system from Millipore (Bedford, MA, USA) and was used to prepare both the aqueous mobile phase and the sample extractant. The extra-virgin olive oil produced in the same agronomic season was purchased from a local market.

Crocin, safranal, and quercetin standards were purchased from Sigma-Aldrich (St. Louis, MO, USA). Kaempferol was purchased from Extrasynthese (Genay, France). Stock solutions were prepared in ethanol.

### 3.2. Optimization Study

Optimization involved the evaluation of three critical variables: temperature, infusion time, and the composition of the medium (extractant). Regarding the extractant, water, extra virgin olive oil, and water/oil combinations in 1:2 and 2:1 (*v*/*v*) proportions were evaluated. The temperature varied at 60 °C, 80 °C, and 100 °C and the infusion time ranged from 10 to 30 min. The total number of experiments, considering all the combinations, was 36 (4 extractant media tested 3 times at 3 different temperatures), and each experiment was performed in triplicate. In the water/oil tests, the two phases were analyzed separately to evaluate the distribution of the target compounds. Therefore, the study resulted in 162 determinations (54 determinations of water and oil single extracts plus 108 determinations corresponding to water/oil assays). Table S1 lists the experiments programmed according to the defined variables. Each extraction was carried out with a sample size of 0.25 g of ground saffron, and the volume of extractant was set at 15 mL. The extractions were carried out in glass tubes using an incubator (Eppendorf ThermoMixer^®^ C, Eppendorf, Madrid, Spain) that allows efficient temperature and vortex control. Once the extracts were obtained, they were stored at −80 °C to avoid any type of alteration. In the case of the water/oil extracts, the phases were previously separated using centrifugation (microcentrifuge Sorvall Legend Micro 21R from Thermo Fisher Scientific, Waltham, MA, USA) for 10 min. The water extracts were centrifuged, filtered, diluted 1:20 (*v*/*v*), and vortexed (shaker from IKA, Wilmington, NC, USA) prior to analysis, while the oil extracts were processed using liquid–liquid extraction with ethanol. For this purpose, ethanol was added to the same volume as the oil. The mixture was then vortexed for 10 min, centrifuged, and 200 µL of ethanol extract (upper phase) was collected and analyzed directly.

### 3.3. LC-QTOF MS/MS Analysis of Saffron Extracts

The extracts were analyzed using LC–MS/MS (high-resolution mode) in data-independent acquisition (DIA) mode. The following compounds were monitored: 2-crocin (cis- and trans-isomers), 3-crocin (cis- and trans-isomers), 4-crocin (cis- and trans-isomers), safranal, picrocrocin, kaempferol, and quercetin. In addition to the flavonoid aglycones, the following derivatives were monitored: kaempferol-O-glucoside, kaempferol-O-diglucoside, kaempferol-O-triglucoside, quercetin-O-diglucoside, and moracetin (quercetin-3-gentiotrioside).

Analysis was performed on an Agilent Technologies LC-QTOF MS/MS 1200 series. Chromatographic separation was performed on a 1200 Series Agilent (Palo Alto, CA, USA) LC system equipped with a C18 reversed-phase column (Zorbax Eclipse Plus C18 HD 3.0 × 150 mm, 1.8 µm) and a guard column (Zorbax Eclipse Plus C18 HD 3.0 × 5 mm, 1.8 µm), using water (phase A) and acetonitrile (phase B) as mobile phases, both with 0.1% of formic acid as an ionizing agent. The following elution gradient was used: from 0 to 1 min, 4% of phase B; from 1 to 6 min, increase from 4% to 40% of phase B; from 6 to 10 min, increase from 40% to 100% of phase B; from 10 to 20 min, maintain at 100% phase B to ensure elution of all sample components. The column was then equilibrated to initial conditions for 13 min prior to the next analysis. The chromatographic flow rate was 0.25 mL/min and the injection volume was 2 µL. The LC system was coupled to a 6540 quadrupole time-of-flight detector (QTOF MS/MS; Agilent Technologies, Santa Clara, CA, USA). Electrospray ionization (ESI) parameters in both negative and positive modes were as follows: nebulizer gas, 40 psi; flow rate and temperature of drying gas (N2), 12 L min^−1^ and 325 °C, respectively; capillary voltage, ±3.5 kV; Q1, skimmer, and octapole voltages, 130, 65, and 750 V, respectively.

In data-independent acquisition (DIA), the acquisition rate was 5 spectra s^−1^ with 3 channels at variable collision energies (CE): 0, 20, and 40 eV, and a cycle time of 1 s per channel (total cycle time 3 s). The acquisition range was 40–1100 *m*/*z* for all channels. The measurement of accurate *m*/*z* values on the 0 eV channel (without fragmentation) in all analyses was ensured using a continuous internal calibration using the signals *m*/*z* 112.9856 (trifluoroacetic acid anion) and 1033.9881 (HP-921, hexakis(1H,1H,3H-tetrafluoropropoxy)phosphazine).

Agilent MassHunter Workstation software (version B7.00; Agilent Technologies, Santa Clara, CA, USA) was used for data acquisition and qualitative and quantitative analysis.

### 3.4. Data Treatment

MassHunter Qualitative Analysis software (version B7.00; Agilent Technologies, Santa Clara, CA, USA) was used to integrate the signals obtained using LC–MS/MS and to construct the data matrix. Metabolite annotation was also performed using MetaboMSDIA (Version 1.00), an R software package developed by the authors, which allows obtainment of multiplexed MS2 spectra for comparison with a homemade MS2 database (Table 1), which includes analytical standards and tentatively identified metabolites based on their characteristic fragmentation [36,37]. Briefly, the software extracts all the signals in the 0 eV channel, which are considered precursor ions, and those in the 20 eV and 40 eV channels, which contain the signals for product ions. Then, considering parameters such as peak shape, intensity, and retention time tolerance, the application associates the precursor ions with their corresponding product ions to obtain an MS2 spectrum for each precursor ion at the different collision energies applied. All experimental MS2 spectra obtained were converted into CEF archives for comparison with those contained in the in-house MS2 library using PCDL Manager software (version B7.00; Agilent Technologies, Santa Clara, CA, USA).

Quantitative analysis was performed by creating calibration models for the available standards (safranal, crocin, kaempferol, and quercetin) in the 1–25 µg/mL concentration range. For those compounds without available standards, quantitation was performed using calibration models of structurally similar compounds. Statistical analysis included the ANOVA test (*p* ≤ 0.05) and pairwise combinations (Tukey HSD) to identify significant differences in the relative concentration of identified compounds.

All extracts were also analyzed according to the method described in the ISO 3632-1:2011 standard for the characterization of color, aroma, and flavor [18]. A Jasco V-730 series spectrophotometer (Madrid, Spain) was used for the UV–vis analysis.

## 4. Conclusions

The results of this study aimed to define the most effective culinary strategy to prioritize the extraction of the main saffron components responsible for color, flavor, and aroma. The compounds showed different distributions depending on the type of phase involved (water, oil, water/oil) and on the infusion time and temperature. Saffron color and aroma are better exploited in the aqueous phase. Temperature plays a role only for color, although good enrichments can be obtained by heating at 100 °C. Regarding the aroma, an oily phase is necessary to significantly improve the safranal enrichment at a temperature not higher than 80 °C; this is in accordance with previous studies. No changes were observed in the concentration of picrocrocin, while heat culinary treatment adversely affects the concentrations of crocins and safranal [26]. The bioactivity of flavonols is better exploited in an aqueous phase, except for kaempferol and moracetin, which are preferably extracted in an oily phase.

Finally, an overestimation of the safranal content obtained using UV–vis was observed. This may be due to interference from other substances present in saffron that absorb at 330 nm, particularly crocins. The determination of safranal using the ISO method did not correlate with that obtained using chromatography. Therefore, LC-MS/MS is preferable to UV–vis for the determination of the three main parameters that define the quality of saffron.

## Figures and Tables

**Table 1:** Parameters for identification, confirmatory analysis, and quantitation of metabolites using LC-QTOF MS/MS.

Compound	Mass	RT *	Formula	Adduct	Precursor Ion	Product Ions
Safranal **	150.105	17.75	C_10_H_14_O	[M + H]^+^	151.1101	91.053, 81.070, 67.054
*trans*-Crocin **	976.375	11.22	C_44_H_64_O_24_	[M − H]^−^	975.3757	327.157, 283.167
*cis*-Crocin **	976.375	13.56	C_44_H_64_O_24_	[M − H]^−^	975.3757	327.157, 283.167
*cis*-Crocin-2	814.326	14.44	C_38_H_54_O_19_	[M − H]^−^	813.3291	327.157, 283.167
*trans*-Crocin-2	814.326	12.38	C_38_H_54_O_20_	[M − H]^−^	813.3291	327.157, 283.167
*cis*-Crocin-3	652.2731	14.23	C_32_H_44_O_14_	[M − H]^−^	651.2651	327.157, 283.167
*trans*-Crocin-3	652.2731	13.49	C_32_H_44_O_14_	[M − H]^−^	651.2651	327.157, 283.167
Picrocrocin	330.1686	10.84	C_16_H_26_O_7_	[M − H]^−^	329.1606	329.160, 149.0972
Kaempferol **	286.0477	14.83	C_15_H_10_O_6_	[M + H]^+^	287.0558	153.016, 121.027
Kaempferol glucoside	448.093	12.68	C_21_H_20_O_11_	[M − H]^−^	447.0933	284.0332, 255.303
Kaempferol diglucoside	610.153	11.42	C_27_H_30_O_16_	[M − H]^−^	609.1377	447.095, 283.0233
Kaempferol triglucoside	772.2065	10.04	C_33_H_40_O_21_	[M − H]^−^	771.1983	446.0853, 284.0319
Quercetin **	302.0426	15.01	C_15_H_10_O_7_	[M + H]^+^	303.0490	153.0199, 127.0527
Quercetin diglucoside	626.148	11.42	C_27_H_30_O_17_	[M − H]^−^	625.1432	463.0843, 301.0327
Moracetin (quercetin 3-triglucoside)	788.2011	11.26	C_33_H_40_O_22_	[M − H]^−^	787.1938	463.0843, 301.0327

* RT, retention time. ** Identification confirmed using analytical standards.

## Data Availability

Data generated and analyzed during this study are provided in full within the article.

## Supplementary Materials

The following supporting information can be downloaded at: https://www.mdpi.com/article/10.3390/molecules29133080/s1, Figure S1. Chemical structures of crocin, crocin-2, and crocin-3. For each of them, cis and trans isomers can be distinguished; Figure S2. Concentration of crocin isomers (µg/mg) as a function of infusion time, extraction medium and temperature; Figure S3. Chemical structure of picrocrocin and safranal; Figure S4. Concentration of kaempferol diglucoside and kaempferol triglucoside (µg/mg) as a function of infusion time, extraction medium and temperature; Figure S5. Concentration of quercetin and derivatives (µg/mg) as a function of infusion time, extraction medium and temperature; Figure S6. Determination of crocin, picrocrocin and safranal by the ISO-3632 method in saffron extracts as a function of infusion time, extraction medium and temperature; Table S1. Experimental design used for the optimization study.
