# Supplementary material for: Distribution of Main Bioactive Compounds from Saffron Species as a Function of Infusion Temperature and Time in an Oil/Water System
Source: Molecules. 2024 Jun 28;29(13):3080. doi: 10.3390/molecules29133080 (PMC11243231; doi:10.3390/molecules29133080)
Supplement: Supplementary file 1 [file molecules-29-03080-s001.zip › molecules-3023661-supplementary.pdf]

Table S1. Experimental design used for the optimization study.

Experiment	Extractant	Temperature	Time	Extract
**01**	Water	60	10	E01
**02**	Water	60	20	E02
**03**	Water	60	30	E03
**04**	Water	80	10	E04
**05**	Water	80	20	E05
**06**	Water	80	30	E06
**07**	Water	100	10	E07
**08**	Water	100	20	E08
**09**	Water	100	30	E09
**10**	Water/Oil 2:1	60	10	E10<br>E11
**11**	Water/Oil 2:1	60	20	E12<br>E13
**12**	Water/Oil 2:1	60	30	E14<br>E15
**13**	Water/Oil 2:1	80	10	E16<br>E17
**14**	Water/Oil 2:1	80	20	E18<br>E19
**15**	Water/Oil 2:1	80	30	E20<br>E21
**16**	Water/Oil 2:1	100	10	E22<br>E23
**17**	Water/Oil 2:1	100	20	E24<br>E25
**18**	Water/Oil 2:1	100	30	E26<br>E27
**19**	Water/Oil 1:2	60	10	E28<br>E29
**20**	Water/Oil 1:2	60	20	E30<br>E31
**21**	Water/Oil 1:2	60	30	E32<br>E33
**22**	Water/Oil 1:2	80	10	E34<br>E35
**23**	Water/Oil 1:2	80	20	E36<br>E37
**24**	Water/Oil 1:2	80	30	E38<br>E39
**25**	Water/Oil 1:2	100	10	E40<br>E41
**26**	Water/Oil 1:2	100	20	E42<br>E43
**27**	Water/Oil 1:2	100	30	E44<br>E45
**28**	Oil	60	10	E46
**29**	Oil	60	20	E47
**30**	Oil	60	30	E48
**31**	Oil	80	10	E49
**32**	Oil	80	20	E50
**33**	Oil	80	30	E51
**34**	Oil	100	10	E52
**35**	Oil	100	20	E53
**36**	Oil	100	30	E54

Crocin

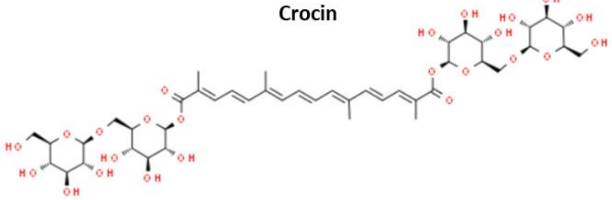

Crocin-2

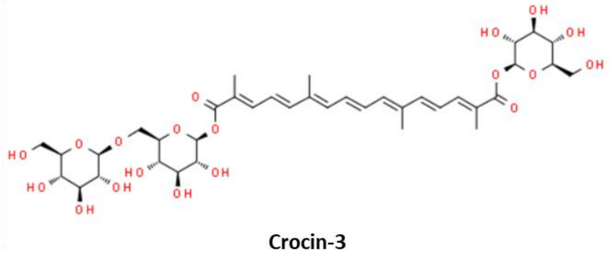

Crocin-3

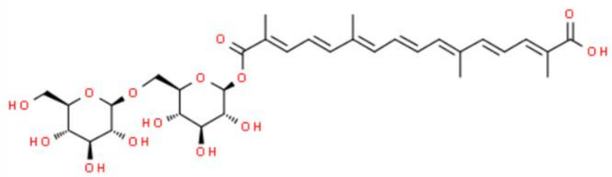

Figure S1. Chemical structures of crocin, crocin-2, and crocin-3. For each of them, cis and trans isomers can be distinguished.

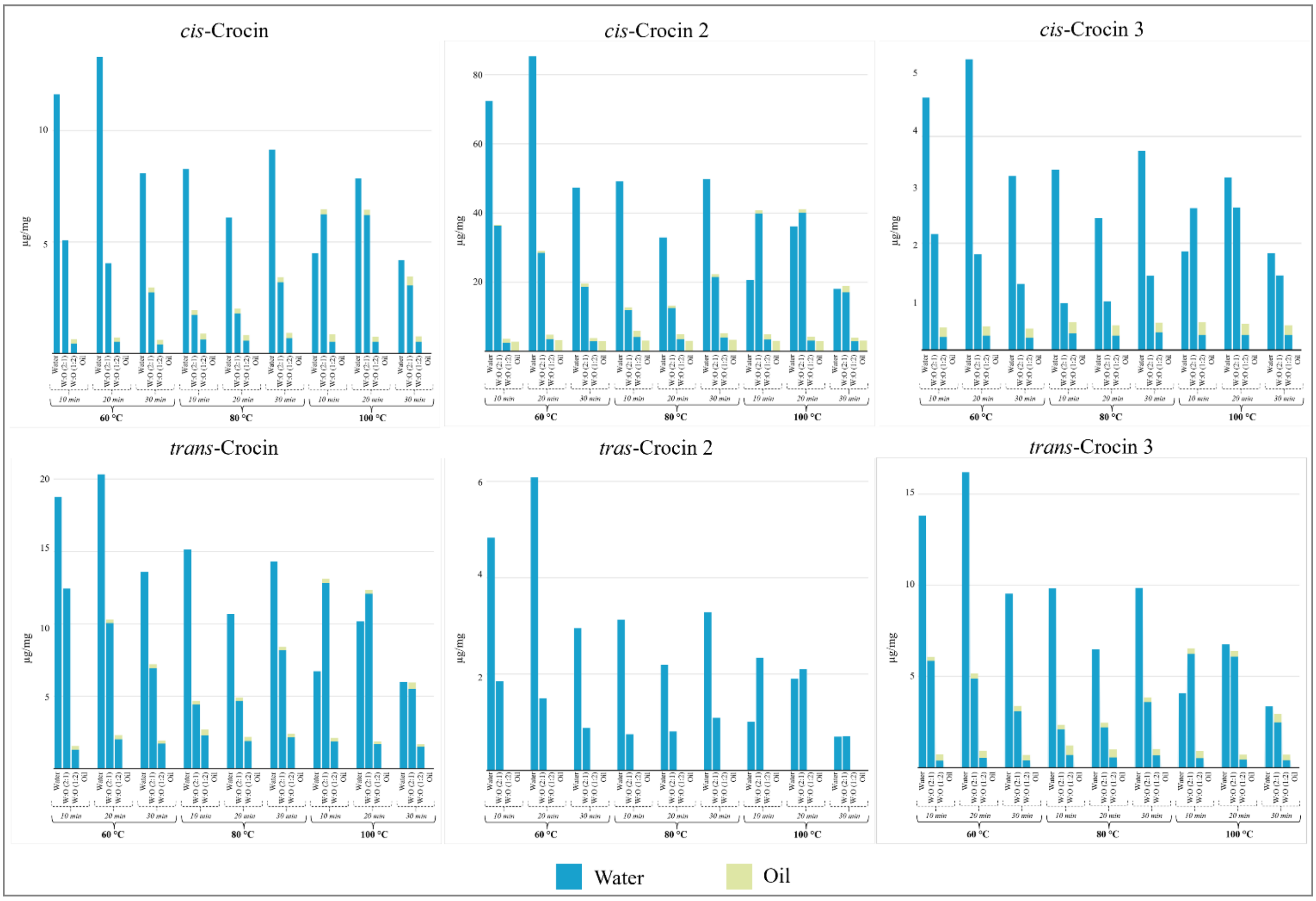

Figure S2. Concentration of crocin isomers (µg/mL) as a function of infusion time, extraction medium and temperature. Concentration variability was always below 15%.

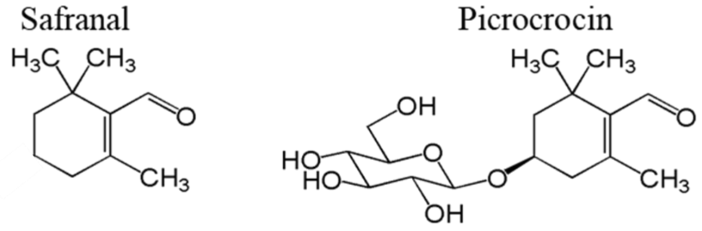

Figure S3. Chemical structure of picrocrocin and safranal.

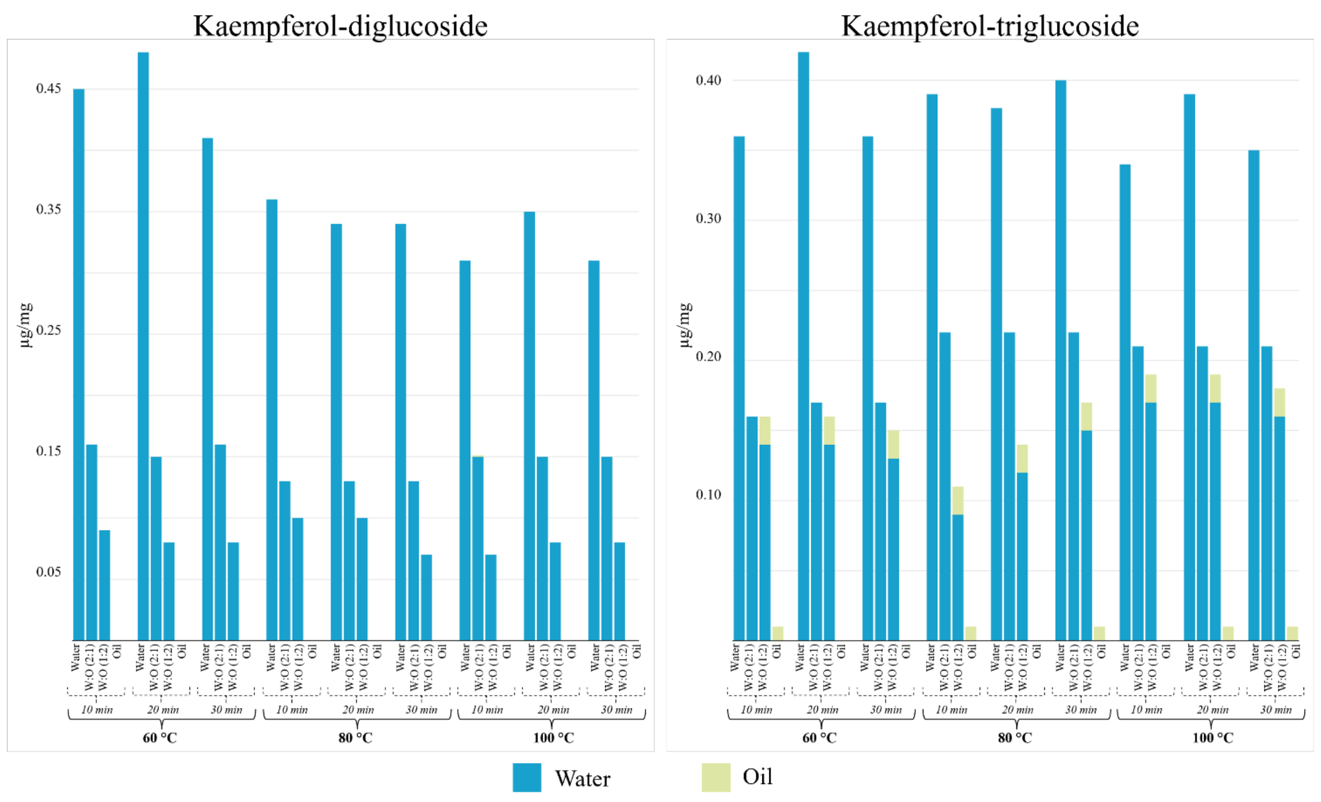

Figure S4. Concentration of kaempferol diglucoside and kaempferol triglucoside (µg/mL) as a function of infusion time, extraction medium and temperature. Concentration variability was always below 15%.

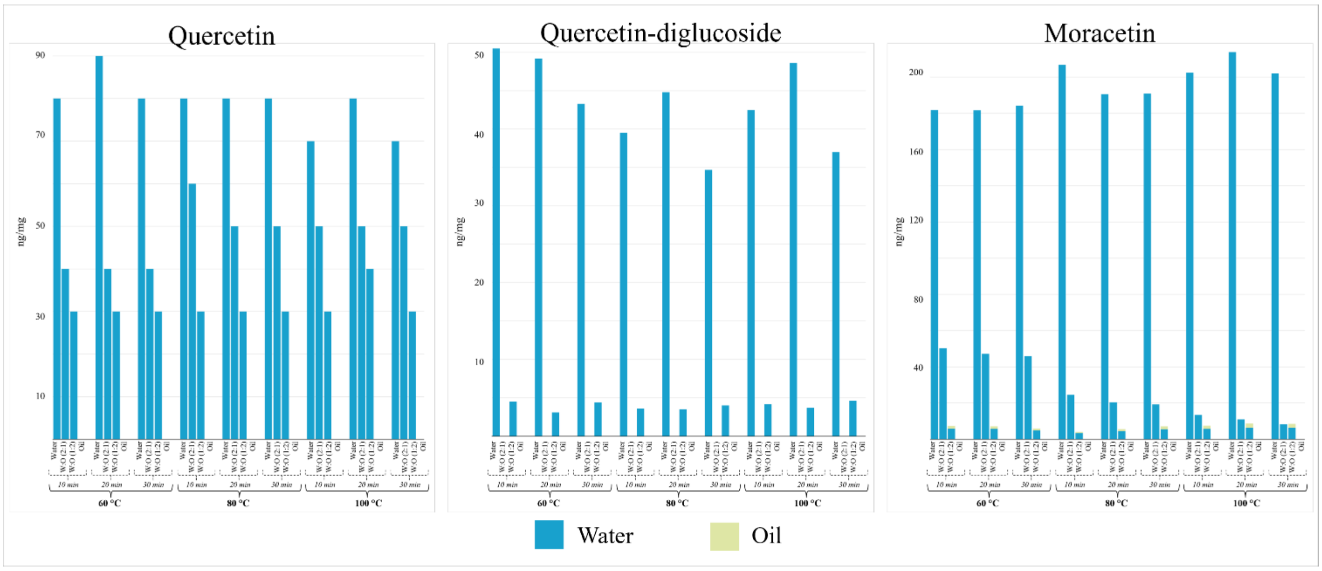

Figure S5. Concentration of quercetin and derivatives (µg/mL) as a function of infusion time, extraction medium and temperature. Concentration variability was always below 15%.

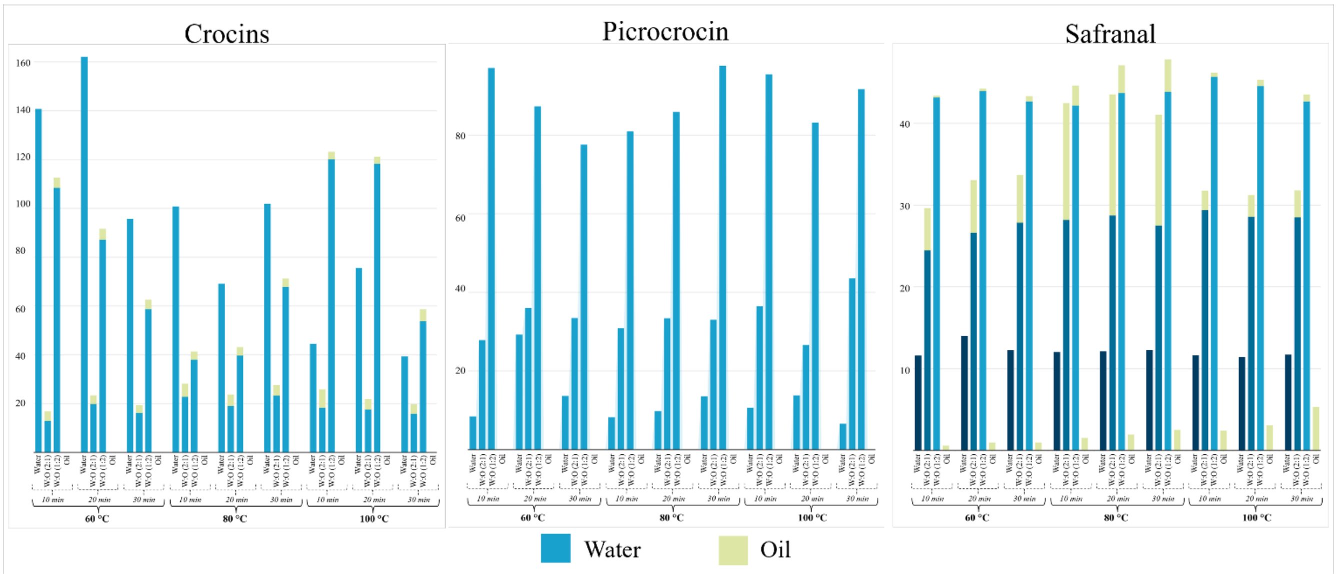

Figure S6. Determination of crocin, picrocrocin and safranal by the ISO-3632 method in saffron extracts as a function of infusion time, extraction medium and temperature. Concentration variability was always below 15%.
